# Polycyclic Aromatic Hydrocarbons in the Snow Cover in the City of Tyumen (Western Siberia, Russia)

**DOI:** 10.3390/toxics10120743

**Published:** 2022-11-30

**Authors:** Dmitriy Moskovchenko, Roman Pozhitkov, Evgeny Lodygin, Marina Toptygina

**Affiliations:** 1 Tyumen Scientific Centre, Siberian Branch of Russian Academy of Sciences, Malygina St., 86, 625026 Tyumen, Russia; 2 Institute of Earth Sciences, Tyumen State University, Osipenko Str. 2, 625003 Tyumen, Russia; 3 Institute of Biology, Komi Science Center, Ural Branch, Russian Academy of Sciences, 167982 Syktyvkar, Russia

**Keywords:** polycyclic aromatic hydrocarbons, snow, atmospheric particulate matter, pollution assessment, source identification, land use

## Abstract

Some of Russia’s large industrial cities are sources of hazardous contamination in the environment. Tyumen is one of the most rapidly developing cities in Siberia due to oil and gas extraction in the northern Tyumen Region. Concentrations of 14 polycyclic aromatic hydrocarbons (PAH)s deposited with the particulate matter (PM) of snow in the city of Tyumen were determined by liquid chromatography. In the background area, the rate of atmospheric particulate deposition was shown to be low, and the mean total content of 14 PAHs had a value of 6.2 ng L^−1^, which is lower than many unpolluted areas on Earth. In the city of Tyumen, the mean content of PM was five times higher and the mean total content of 14 PAHs was twenty times higher as compared to the background. The contents of chrysene, benzo(k)fluoranthene, and benzo(a)pyrene were increased by multiples of 78, 77, and 32, respectively. The rates of ∑14 PAH deposition with airborne PM over the winter ranged from 1.1 to 65.5 μg m^−2^. Calculations of BaP toxic equivalent showed maximal toxicity within the transport zone. Both analysis of spatial distribution and diagnostic ratios showed that the PAHs were mainly from coal combustion and vehicle emissions.

## 1. Introduction

Research on polycyclic aromatic hydrocarbons (PAHs) in the environment is very important due to their carcinogenic properties, high toxicity, stability, and tendency for accumulation. Hazardous health impacts are the major motivation for studying atmospheric PAHs [1]. There are 16 PAHs included in the list of Priority Pollutants by the United States Environmental Protection Agency. Atmospheric deposition is a major source of PAHs in surface soil and street dust [2]. PAHs occur in the atmosphere in the form of gases and aerosols. Particulate-bound PAHs are washed out from the atmosphere via physical processes controlled by size, number density, and solubility of aerosol, along with meteorological conditions and microphysics [3].

The PAHs in fresh snow and snowpack could reflect the PAH levels in atmosphere [4]. Snowpacks, particularly those in the urban environment, act as a reservoir by accumulating PAHs during the winter period [5]. In temperate latitudes, concentrations of PAHs increase during the winter due to increased emissions from thermal power stations and the prevalence of anticyclones characterized by subsiding air. In addition, snow can absorb PAHs more efficiently than rain [6]. As a result, in the winter, atmospheric PAH concentrations in the temperate regions are generally higher than in the summer [7,8]. For example, winter aerosols in Novosibirsk City (Western Siberia) are more toxic than summer aerosols [9].

In Russia, snow cover is present for periods of 4–7 months each year, which leads to the accumulation of pollutants. Consequently, the winter-long accumulated release of PAHs produces high concentrations of PAHs during the snowmelt period, causing a shockload to receiving waterbodies [5].

Despite the fact that snow cover is present for significant periods of time in most regions of Russia, snow has rarely been used in assessments of emissions of organic pollutants. There are only a few papers on this topic, which include studies on PAHs in snow in the following cities: Moscow in the European part of Russia [10,11,12], Bratsk and Shelekhov in Eastern Siberia [13,14], Barnaul in the south of Siberia [15], Khabarovsk and Blagoveshchensk in the Far East [16,17], and Arkhangelsk, a city in the north of the European Russia [18]. Background concentrations of PAHs in snow have been determined in the northeast of the European part of Russia [19] and Western Siberia [20]. However, for the vast majority of large regional centers, studies are completely absent.

In this study, we assessed the PAH concentrations within the particulate matter (PM) phase of snow in the city of Tyumen, which is a large and actively developing administrative center. The rapid development of Tyumen has been primarily due to the discoveries of numerous oil deposits in the north of Western Siberia during the second half of the 20th century. Tyumen is a large transportation hub. Transport flows pass through it, heading north towards hydrocarbon reservoirs. Significant levels of soil pollution by benzo(a)pyrene had previously been reported in Tyumen in the late 1990s [21]. Tyumen now has a number of industrial plants producing machinery, equipment and building materials, petroleum refineries, and power stations. A study on the 12 PAHs revealed that soils of Tyumen are polluted within low-residential and transport areas and that the low molecular weight (LMW) PAHs anomaly is fixed in close proximity to the oil refinery [22].

Tyumen is currently experiencing rapid growth. Since 2005, the area of the city increased threefold and the population 1.5 times [23]. Some plants and factories have been recently built. Large enterprises such as Tyumen electrometallurgical plant and Antipinsky Refinery are among them. Therefore, the number of PAH sources keeps growing, which justifies the need to study the ways of admission of PAHs, their sources, and their hazard to public health. 

The objectives of this study were (1) to assess the PAHs in the PM phase of snow within the city of Tyumen; (2) to identify their sources; (3) to evaluate PAH concentrations and toxicity in areas of different land uses. 

Our decision to study the PM phase of snow was dictated by the fact that atmospheric PM deposition is the main source of airborne pollutants, including PAHs. Most of the PAHs with low vapor pressure in the air are adsorbed on PM particles, due to the fact that even though PAHs show low solubility in water, they are highly lipophilic [24]. A number of studies have indicated that the majority of the harmful compounds in the atmosphere exist in fine particulate matter [25,26]. About 80–90% PAHs in snowmelt samples are associated with solids [5].

## 2. Materials and Methods

### 2.1. Study Area and Sampling

Tyumen is located in the southwestern part of the West Siberian Plain, at the southern margin of the taiga zone. The climate of Tyumen is cold humid continental. Average monthly temperatures range from −22 °C in January to +17 °C in July. The average total precipitation is estimated as 480 mm year^−1^ [27]. On average, stable snow cover is present for five months of the year, from early November to early April. The cold period is characterized by a predominance of southern and western winds, which carry pollutants to the north and northeast.

The city also currently has 385 thousand motorized vehicles in use and a network of roads totaling 1241 km in length [22,23]. Major transport routes pass through Tyumen to northern regions of Western Siberia, where the largest oil and gas fields are located. Exhaust gases from motorized vehicles make up more than 80% of the total emissions of pollutants into the atmosphere within the city [28]. Traffic on the busiest roads reaches 8.1 thousand vehicles per hour [29].

Snow samples were taken 18–20 February 2020. The snow cover settled in mid-November. The samples were taken 102 days thereafter. A total precipitation of 106 mm was recorded over the period from November 2019 to February 2020. There were 44 snowy days during that period [30]. The long duration of snow cover and many precipitation days allowed us to accurately estimate the level of atmospheric deposition of particulate matter of snow during the winter.

**Figure 1:**
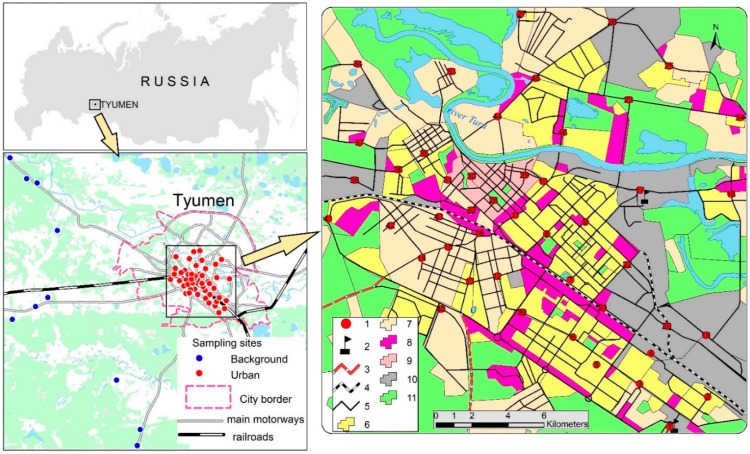
Sampling sites and land-use areas within the city of Tyumen, Russia: 1, sampling sites; 2, power plants; 3, main federal roads; 4, Trans-Siberian Railway; 5, main city roads; 6, high-rise residential areas; 7, low-rise residential areas; 8, business and public facilities areas; 9, historical center; 10, industrial zones; 11, recreation and unbuilt.

Snow was sampled from within the Tyumen city and from background sites at distances of 20–35 km to the west and southwest from the city (Figure 1). By taking into account that winds from the south prevailed during the study period, the locations of background sampling sites excluded any contamination of snow by pollutants from Tyumen. During sampling, the land-use type of each surveyed area was taken into account. The functional zones (different land-use areas) within Tyumen are not clearly separated, but form a mosaic pattern of industrial, residential, commercial, and recreational areas within the city. However, different types of land use were recorded at the sampling sites as follows:

(1) the historical center with buildings that have existed since the 17th century and are now used by social and administrative organizations;
(2) low-rise residential areas that have existed since the 19th century;
(3) high-rise residential areas that have been constructed within a period from the 1950s to the present time and that now house the major part of the Tyumen population;
(4) business and public facilities areas;
(5) industrial zones;
(6) transport zones affected by road traffic (located between main roads and buildings of various usage).

Sampling within transport zones was carried out at distances of more than 15 m from roads in order to exclude direct contamination by road dust.

Samples of snow were taken using a VS-43 snow gauge, which is a widely used instrument for meteorological observations in Russia. The samples were taken as a vertical profile from the top to the bottom of the snow bank, except for the lowest 3 cm in order to exclude contamination with soil particles. The sampled snowpack was visibly undisturbed by any human activity. At the same time, we measured the depth of snow cover and snow density.

Samples were placed in 24 dm^3^ plastic containers with lids, which were washed with distilled water prior to sampling. Closed containers with samples were immediately transported to the laboratory. In total, 46 samples were taken from the city of Tyumen and 8 samples from background sites. A detailed description of the sampling sites is presented in Supplementary Materials Table S1.

### 2.2. Sample Preparation and Analyses

After the snow samples were melted at room temperature, they were filtered through pre-weighed glass microfiber filters of 0.45 µm pore size (Sartorius, Goettingen, Germany) using a vacuum filtration device. The filters were dried at 95 °C and weighed again for determination of the mass of trapped solids. In melted samples, we measured pH values using an HM-500 HydroMaster ionometer, and total dissolved solids (TDS) was determined by using a COM-100 conductometer (both HM Digital, Khushkhera, India). Then, 14 U.S. Environmental Protection Agency priority-listed PAHs were detected in snow solid phase samples: naphthalene (NaP), fluorene (Flu), phenanthrene (Phe), anthracene (Ant), fluoranthene (Flt), pyrene (Pyr), benzo(a)anthracene (BaA), chrysene (Chr), benzo(b)fluoranthene (BbF), benzo(k)fluoranthene (BkF), benzo(a)pyrene (BaP), dibenzo(a,h)anthracene (DahA), benzo(ghi)perylene (BghiP), indeno[1,2,3-cd]pyrene (IcdP).

Determinations of PAHs were carried out in the Chromatography Center at the Institute of Biology of Komi Science Center of the Ural Branch of the Russian Academy of Sciences. PAHs were extracted at room temperature using a 1:1 hexane–acetone mixture following guidelines of the U.S. Environmental Protection Agency [31]. Impurities were removed from the PAH fraction using the column chromatography method with silica gel [32]. Qualitative and quantitative analyses of PAHs were conducted by the use of a Lumachrom chromatograph (Lumex, Saint Petersburg, Russia) via reversed-phase liquid chromatography in a gradient mode and spectrofluorimentric detection. Chromatography was performed at a temperature of 30 °C using a Supelcosil™ LC-PAH column (5 µm particle size) (Supelco, Bellefonte, PA, USA). Identification of each PAH was performed by comparison of the fluorescence spectrum from the analyzed column with standard spectra of PAHs. The quantitative analysis of polyarenes was carried out with external standard. The accuracy of this method was assessed by applying the above-described analytical procedure to the standard PAH-containing sediment (Standard Reference Material^®^ 1944 New York/New Jersey Waterway Sediment), which gave satisfactory results. Analysis of three laboratory blank filters demonstrated that no significant footprints for PAHs were found.

### 2.3. Calculations and Data Analysis

A statistical analysis was conducted with the use of Statistica 10.0 software (StatSoft, Tulsa, OK, USA). All PAHs were divided into two groups according to the number of aromatic rings: (1) low molecular weight (LMW), two to three-ring PAHs (NaP–Flt); (2) high molecular weight (HMW), four to six-ring PAHs (Pyr–IcdP). Values below the detection limit were counted as zero.

The concentration data were used to calculate the loading or deposition rates of PAHs stored in the snow with the use of the following equation:

m = Cn × W(1)

where m is the loading rate (ng/m^2^), Cn is the concentration of each target analyte in the snowmelt sample (ng L^−1^), and W is the water reserve in snow, L m^−2^.

The PAH deposition rate per 1 m^2^ per day was calculated by means of dividing the total PAH content by the period (102 days) of the presence of snow cover.

In order to evaluate the degree of pollution, we calculated contamination factor (CF) values as follows [33]:

CF = Cn/Cb(2)

where Cn is the measured concentration of the element in the sample and Cb is the background value of the element concentration.

The contamination factor indicates the pollution degree but does not take into account the fact that different PAHs have diverse toxic and cancerogenic effects. The health risk associated with inhalatory exposure to PAHs either in the occupational atmosphere or in outdoor air is commonly assessed on the basis of benzo(a)pyrene (BaP) concentrations [34]. BaP is estimated to be the major contributor to the cancer risk of PAHs [35]. Most PAHs are not as highly toxic as BaP [36]. The evaluation of the benzo(a)pyrene equivalent concentration (BaPeq) was defined as the carcinogenic and toxic potency of the individual PAHs relative to BaP. The BaPeq approach is believed to take into account the total carcinogenic potency arising from exposure to a mixture of various PAH compounds with different toxicities [37]. The concentrations of each PAH were multiplied by their toxic equivalency factor TEF values and expressed as the BaP toxic equivalent quantity for the mixture of PAHs to assess the toxicity of each sampling site:

(3) $$\mathrm{BaPeq}=\sum_{i=1}^{n}\mathrm{PAHi}\times \mathrm{TEFi}$$

where PAHi is the concentration of individual PAHs (ng L^−1^), and TEFi is the toxicity equivalency factor of individual PAHs, according to [36].

Russian environmental standards on PAHs regulate the mass fraction of benzo(a)pyrene in the soil (20 ng g^−1^). We calculated the mass fraction of BaP in solids (ng g^−1^) by dividing the content of the matter by dust weight in the sample in order to determine the risk of atmospheric deposition of PAHs and its impact on the soil composition. The criterion of the danger of soil pollution is the degree of excess of BaP concentration in soils with respect to MPC, or the toxic unit (TUBaP), calculated as follows:

TUBaP = Ci/MPC(4)

where Ci is the BaP concentration in snow particulate matter (ng g^−1^) and MPC is the BaP maximum permissible concentration [22].

PAHs diagnostic ratios were used for identifying and assessing the appropriate pollution emission source. Diagnostic ratios of PAHs are mainly used for distinguishing between combustion-related sources [38]. In this study, we calculated such diagnostic ratios as Flt/Pyr and LMW/HMW. According to [39], the Flt/Pyr ratio is well-suited to the analysis of sources of PAHs in the snow, as opposed to other popular ratios, such as Phe/Ant or BaA/(BaA + Chr). The LMW/HMW ratio has been successfully applied to analyze the admission of PAHs in the snow cover [16,40].

The spatial distribution of pollutant concentrations was assessed using ArcGIS 10.6.1 software (Esri, Redlands, CA, USA), where the Raster Interpolation tools create isolines using inverse distance weighting.

## 3. Results and Discussion

### 3.1. Physical and Physico-Chemical Properties of Snow

The data obtained on snow cover characteristics including particulate matter (PM) contents and deposition rates are presented in Table 1.

Snowmelt water from the background sampling sites had an acidic reaction, which is typical for snow cover within the taiga zone of Russia. The mean pH value (4.7) in the studied samples coincided with that in meltwater from the taiga zone of the Komi Republic (the European part of Russia) [41], but it was lower than the mean pH (5.3) in meltwater in the Khanty–Mansi Autonomous Okrug (Western Siberia) [42]. Snow densities ranged from 0.16 to 0.30 g cm^−3^. The content of PM in snowmelt water samples from the background sites varied from 4.0 to 10.9 mg L^−1^, with a mean of 7.5 mg L^−1^. In snow from unpolluted ecosystems in remote regions of Arctic, contents of insoluble solids vary from 0.2 to 3 mg L^−1^ [43]. Therefore, the content of PM in snowmelt water around Tyumen was higher than that in remote places on Earth located far from industrial regions, which indicates that dust aerosol emissions from various local and distant sources are present within the Tyumen Region. Numerous local sources of dust emissions into the atmosphere are associated with human settlements. There also are erosional sources including roadside zones that are free from snow due to the application of road salt and other deicing agents.

The snowpack within the city of Tyumen had a mean depth of 26 cm, which was significantly lower than that within the background area (41 cm), see Table 1. The city was also characterized by a higher density of snow, which resulted from higher temperatures and melting. Generally, cities are known as ‘urban heat islands’, from which the snow cover disappears sooner than from surrounding areas. The content of PM phase in snowmelt waters of Tyumen ranged from 9.4 to 121 mg L^−1^ with a mean value of 37.5 mg L^−1^, which is five times higher than that in the background area. The city also differed from the background area by the occurrence of samples with neutral and weakly alkaline reactions, with a difference of 1.6 between the mean pH values in Tyumen and in the background area (Table 1). The alkalinization of snow observed in Tyumen was more significant than such trends observed in other Russian cities. For example, the mean pH of Moscow meltwaters was increased by only 0.4 as compared to background [44]. The alkalinization of snow was indicative of the presence of carbonate dust particles, which originate from building materials.

### 3.2. PAH Content

**Figure 2:**
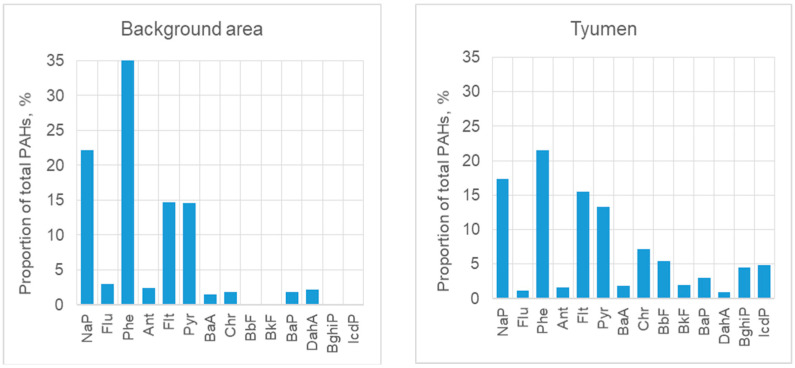
PAH distribution patterns of atmospheric depositions from the background area and Tyumen, with the percentage of each individual substance of the total PAH concentration (C/Ctotal, %).

Statistical parameters of PM-phase PAH concentrations are shown in Table 2. The total content of 14 PAHs in the PM phase of samples from the background area had a mean value of 6.2 ng L^−1^. Low molecular weight PAHs such as phenanthrene (Phe), naphthalene (NaP), and fluoranthene (Flt) were predominant (Figure 2). NaP are considered to be associated with petroleum sources but not combustion [45,46]. Among high molecular weight (HMW) PAHs, pyrene (Pyr) was the most abundant. The results demonstrate that low-molecular-weight PAHs (Phe, NaP, Flt) prevail in solids in background areas, while the content of high molecular weight PAHs is 3.5 times lower. Benzo(a)pyrene (BaP) was found in only half of all samples, and its concentration had a maximal value of 0.35 ng L^−1^, which is much lower than its maximal permissible concentration of 1 ng L^−1^, according to Russian standards. Concentrations of other HMW PAHs were below the detection limit in most samples.

We compared the results obtained with the data on other background (unpolluted) places on Earth. The mean total concentration of 14 PAHs (6.2 ng L^−1^) in the background area of Tyumen is slightly lower than that (8.7 ng L^−1^) in the taiga zone of the European part of Russia [19]. It is much lower than the total concentrations of 13 PAHs (from 34.8 to 59.6 ng L^−1^) in the Russian Far East [16] as well as the total concentrations of 16 PAHs (from 20.45 to 60.57 ng L^−1^) in the glaciers of Tibet [47]. Only in snow from the Alps was the range of total concentrations of 24 PAHs (0.5–8.4 ng L^−1^) [48] slightly lower than the concentration range (1.7–10.9 ng L^−1^) found in the study area. The low total content of PAHs in the background area of Tyumen is probably connected with the significant distance of sampling sites from PAH sources.

In an urbanized area, the total concentration of PM-phase PAHs ranged from 17.8 to 1018.9 ng L^−1^. Such significant variation is indicative of multiple sources that do not reach out to the entire city but affurect only local areas. The average PAHs content (123 ng L^−1^, see Table 2) is 20 times higher than that in the background area. Concentrations of total PAHs in snowpack were significantly different among urban and background locations (*t*-test, *p* < 0.05).

Mean concentrations of individual PAHs decreased in the following order: Phe > NaP > Flt > Pyr > Chr > BbF > IcdP > BghiP > BaP > BkF > BaA > Ant > Flu > DahA. The most abundant substances (Phe, NaP, and Flt) are 2–3-ring PAHs that have predominantly petrogenic origin. The high proportion of LMW PAHs indicated the petrogenic origin of PAHs, either due to fuel or petroleum spills [22]. The sum of Phe, Flt, and Pyr corresponded to about half of the total PAHs, which agrees with the previously reported data from cities of Eastern Siberia [49]. Snow of the Moscow Region has been characterized by the predominance of Phe and the absence of Flu [50]. Most reports have indicated that LMW PAHs and four-ring PAHs were the main components of PAHs in the atmosphere [51]. In general, the predominance of LMW and 4-ring PAHs established by us in Tyumen corresponds to the common distribution of PAHs in atmospheric precipitation.

In the soils of Tyumen, contents of PAHs decreased in the following order: Phe > Pyr > Flt > BghiP > BbF > Ant > BaP > BaA > Flu > BkF > DahA > NaP [22]. A comparison with our data shows that the content of NaP in soils is low in contrast to the particulate matter of snow. NaP is the most unstable in soils, and mainly due to its low molecular weight, it is characterized by high volatility and better solubility; as a result, it has a high migration capacity and is actively decomposed by microorganisms [52]. Thus, the naphthalene transformation in soils results in the decreased concentration of this compound. Other PAHs are found in comparable proportions in the PM phase of snow and in soils.

The city, as compared to the background area, was characterized by decreased percentages of NaP, Flu, Phe, and Ant and increased percentages of Flt, Chr, BbF, BkF, BaP, BghiP, and IcdP (Figure 2). Flt and Chr were indicative of the influence of motorized vehicles, because these two substances are known as markers of pollution by exhaust gases [53]. The city was characterized by the appearance of 6-ring PAHs such as benzo(ghi)perylene (BghiP) and indeno[1,2,3-cd]pyrene (IcdP), which are indicators of wood-burning [54].

### 3.3. Source Identification

Anthropogenic sources of PAHs are subdivided into petrogenic and pyrogenic. Natural objects can contain a combination of hydrocarbons from different sources, which complicates the source identification [55,56]. The genesis of PAHs is usually determined by using certain ratios. The most accurate methods of identifying petrogenic sources are based on ratios of Flt/(Flt + Pyr), BaA/(BaA + Chr), and Ant/(Ant + Phe) [39]. Their application is based on the prevalence of certain isomers in different anthropogenic processes.

The diagnostic role of the above ratios has been proved through studies of such natural environments as bottom sediments, soils, and road dust. In particular, based on Flt/(Flt + Pyr) and Ant/(Ant + Phe) ratios, motor transport is considered the primary source of PAH admission in the soils of the city of Tyumen [22]. Nevertheless, the variety of PAH transformations in different natural conditions regarding photochemical breakdown and microbial decay reduces the informative value of diagnostic ratios [39,57].

The particulate matter resides in the snow at negative temperatures, and microbiological activity is, therefore, suppressed. Photochemical reactions in the depth of snow cover proceed more slowly than on the surface. This is why the conditions for PAH transformations in the snow are substantially different from those typical of soils and road dust, which makes the use of diagnostic PAH ratios difficult. The study of PAHs in the snow has demonstrated that such ratios as Phe/Ant and BaA/(BaA + Chr) are practically unsuitable for assessing the source of pyrogenic PAHs in the snow cover [39]. Flt/Pyr and BbF/BghiP ratios performed the best, indicating the sources of PAHs in the northern region of West Siberia [20]. Since the content of BghiP in the particulate matter of Tyumen snow was often below the detection limit, we considered the use of BbF/BghiP ratio as impractical. This is why, along with the Flt/Pyr ratio, we applied the LMW/HMW ratio, which has been repeatedly used in studies on PAH contents in the snow [16,40].

**Figure 3:**
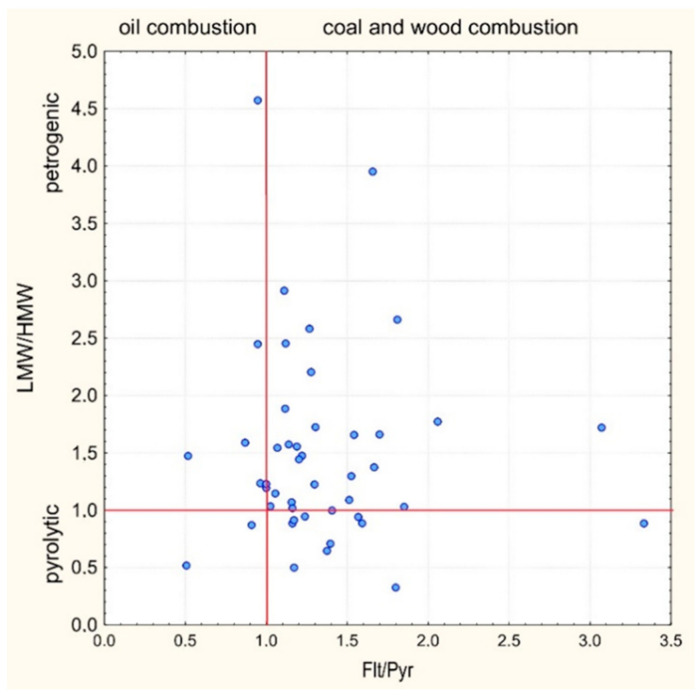
Cross-plots of the values of LMW/HMW against the values of Ft/Pyr.

The results of calculations of PAH diagnostic ratios are presented in Figure 3. According to [39], the value of Flt/Pyr < 1 if the admission of PAHs is associated with the burning of hydrocarbons. In 9 of the 46 analysed samples (i.e., 20%), the Flt/Pyr ratio in most samples had values below 1 and hence considered as an indicator of hydocarbon combustion processes. A significant number of samples is characterized by Flt/Pyr ≈ 1 (see Figure 3), which is indicative of a combined effect of flaring oil products and solid fuels (coal and wood). The highest values were observed in the peripheral southwestern part of the city, where the composition of the atmosphere is affected by air transport from the suburbs, where firewood and coal are widely used for stove heating.

LMW/HMW ratio values >1 indicate that sampling sites were contaminated mainly by petrogenic PAHs, while values <1 are indicative of pyrogenic sources [58]. The higher molecular weight PAHs dominated in the pyrolytic PAH contamination distribution [59,60]. Based on LMW/HMW ratio, only 22% of PAH samples clearly originated from pyrogenic sources (LMW/HMW < 1). Those samples were mainly taken from within the transport zone. In general, the mean value for the transport zone is LMW/HMW = 1 (Table 3), which evidences a combined effect of pyrogenic and petrogenic sources

However, it should be borne in mind that the threshold value LMW/HMW = 1 distinguishing the predominance of either pyrogenic or petrogenic sources was determined by analyzing the content of PAHs in bottom sediments [58]. Threshold levels for snow may be different. Data published elsewhere show LMW/HMW < 1 even in the largest metropolises with numerous PAH sources, which speaks to their non-pyrogenic origin. For instance, the total content of LMW PAHs in Moscow snow overtops the content of HMW PAHs [20]. These results contradict the conclusions by [61] about the predominance of HMW PAHs in Moscow soils. In the same manner, [16] argues that ∑LMW PAHs in the snow surpassed ∑HMW PAHs at most sample sites in Khabarovsk. As we have mentioned earlier, the ratio of PAH isomers may significantly differ in soils and snow. The different hydrophilic nature of LMW and HMW can explain the change in their ratio in the snow, which affects the intensity of the deposition of LMW and HMW with precipitation. HMW PAHs are relatively immobile because of their large molecular volumes and their extremely low volatility and solubility [62]. The lightest PAHs can easily be captured by water droplets during washout [63].

It is known that the composition of PAHs is unstable throughout the year. An increase in the air temperature causes a complete or a partial transformation of LMW and medium-molecular-weight (MMW) PAHs (phenanthrene, fluoranthene, pyrene, and chrysene) from the particulate phase (aerosol) into the gaseous phase of the atmosphere by means of photodestruction, biological degradation, and removal through evaporation and leaching; hence, the PAH concentrations in aerosols reach their minimal values during the summer [64,65,66]. Consequently, during the winter, the concentrations of LMW PAHS reach their maximum, as was observed in the PM phase of Tyumen snow that was dominated by Phe, NaP, Flt, Pyr, and Chr (Table 2).

It is necessary to state that threshold levels of diagnostic ratios of PAHs require clarification in snow studies.

### 3.4. Spatial Distribution and Land-Use Effects

As demonstrated in Table 3, different PAH sources dominate different functional zones of Tyumen. Based on LMW/HMW and Flt/Pyr ratios, the transport zone is foremost exposed to pyrogenic sources. The intensity of PAH deposition is the highest there, too. Total contents of PM-phase PAHs in different land-use areas decreased in the following order: transport zones (mean value 234 ng L^−1^) > historical center (113) > low-rise residential areas (101) > industrial areas (74) > high-rise residential areas (63) > business and public facilities areas (59). Within the transport zone, the total of 14 PAHs was approximately three times higher than values of this parameter within the high-rise residential areas as well as industrial and business zones. LMW PAHs predominate in all zones.

**Figure 4:**
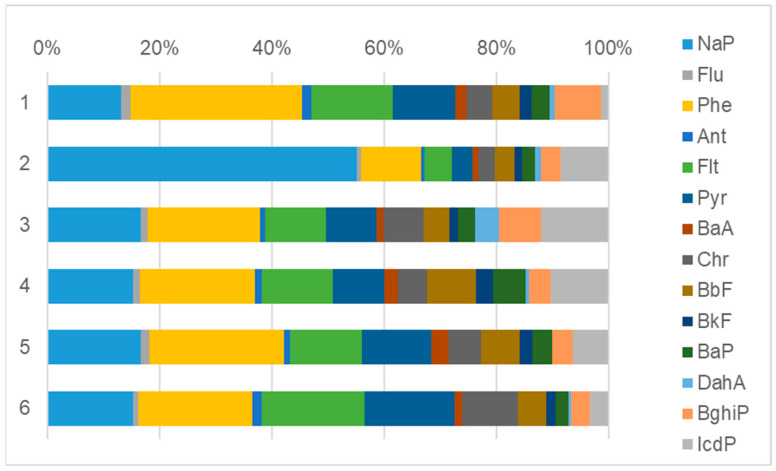
Contributions of individual PAHs within the following land-use areas of Tyumen: 1, historical center; 2, low-rise residential areas; 3, high-rise residential area; 4, business and public facilities areas; 5, industrial zones; 6, transport zones.

There were certain differences in the proportions of PAHs in functional zones of Tyumen city. The historical center had a high percentage of Phe, the low-rise residential areas had a high percentage of NaP, and the transport zone was characterized by increased concentrations of Flt, Chr, and Pyr (Figure 4). Relatively high percentages of Phe (in the historical center) and NaP (in the low-rise residential areas) are associated with the use of coal and wood for house heating, as these PAHs are found in soot resulting from coal combustion [55,67]. The influence of the long-standing use of coal and firewood to heat dwellings shaped PAH anomalies in soils of the single-stage residential and central areas of Tyumen [22].

In the transport zone, increased percentages of Flt and Chr correspond to the established concept that these compounds are emitted with exhaust gases [53]. The increase in the Pyr content also occurs under the impact of motorized vehicles, as their exhaust gases contain ten times more pyrene than benzo(a)pyrene [68]. In the center of Erzurum city (Turkey), intense traffic areas have also been characterized by increased Pyr concentrations in snow [69]. However, the polyarene composition had only minor variations in different functional zones of Tyumen, except for the low-rise residential aread. This can be explained by the absence of clear divisions between the functional zones of Tyumen and a relatively homogeneous composition of the city air, where particulate matter is suspended. A similar situation has been observed in Saint Petersburg (Russia), where levels of soil pollution in recreational and public zones are shown to be comparable with those in industrial and transport zones as a result of the dispersion of PM-phase contaminants in the air [70].

Differences in the intensity of deposition of PAHs and in the correlation of certain isomers determine differences in toxicity. The mean values of BaPeq were dependent on the land use of the territory, ranging from 4.1 ng L^−1^ in the industrial zone to 10.5 ng L^−1^ in the transport zone (Table 4). The most toxic samples with the maximum BaPeq were found near the roads with the busiest traffic.

BaPeq values in all of the sample sites were obviously lower than guideline value recommended by the US EPA (200 ng L^−1^) but higher than the Chinese CEPA value for the surface water (2.8 ng L^−1^) [71,72]. The greatest contributions to toxicity were made by BaP (53%), DahA (18%), and BbF (9.9%). According to our results, the entry of snowmelt water into rivers used to supply water to Tyumen during the thawing season can be hazardous and cause population health risks.

**Figure 5:**
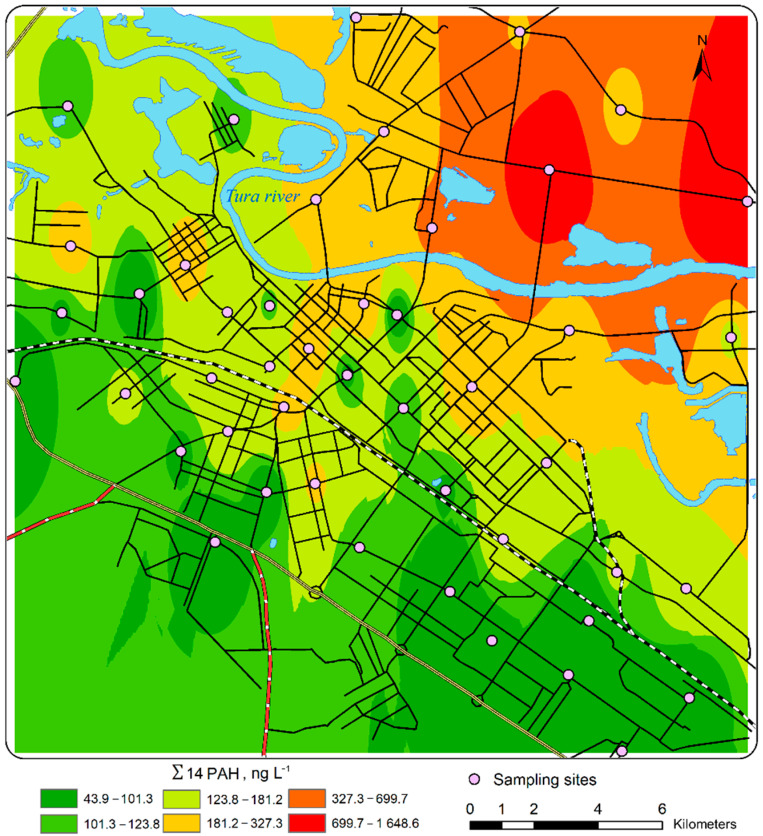
The distribution of ∑14 PAHs in the PM phase of snow in Tyumen.

The spatial distribution of the total content of 14 PAHs is shown in Figure 5. The maximal pollution was identified in the northern part of the city, where ∑14 PAH concentrations varied from 180 to 700 ng L^−1^. In this part of the city, a high concentration of airborne dust particles resulting primarily from intensive road traffic has also been reported in a recent study on PM_2.5_ (particles 2.5 microns and below) and PM_10_ (10 microns and below) in the atmospheric surface layer of Tyumen [73]. The predominance of southern winds is probably an additional factor contributing to the high concentration of airborne pollutants in the northern part of the city, while its southern part remains relatively clean.

### 3.5. Contamination Levels and Comparison with Other Data

**Figure 6:**
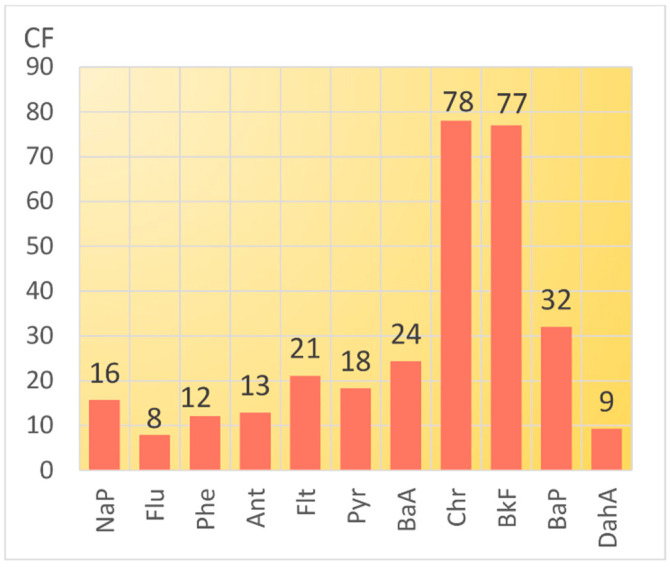
Mean values of contamination factor (CF) of PAHs in snow samples from Tyumen.

As compared to the background areas, the content of certain isomers in Tyumen snow is 7.9–78.0 times higher (Figure 6). The most significantly increased concentrations of chrysene, benzo(k)fluoranthene, and benzo(a)pyrene corresponded to the CF values of 78, 77, and 32, respectively.

The mean concentration of BaP in particulate matter of snow amounted to 126.7 ng g^−1^ (the highest value was 676.8 ng g^−1^). Calculation of TUBaP showed that BaP concentration did exceed the established MPC (20 ng g^−1^) in 92% of samples. As earlier discovered by Konstantinova et al. [22], BaP contents in Tyumen topsoils exceeded the established MPC in 77% of samples and BaP concentration changed from 9.9 ng g^−1^ (high-rise residential areas) to 15.2 ng g^−1^ (low-rise residential areas), while BaP concentration in road dust was slightly higher and amounted to 26.1 ng g^−1^ [23]. Thus, BaP concentration in insoluble solids of snow is by an order of magnitude higher than in soils. Consequently, insoluble solids deposited with snow are one of the primary sources to enrich urban soils with benzo(a)pyrene. This illustrates the need for shoveling snow and transporting it to special storage areas.

We compared the content of PAHs in Tyumen snow with that in other cities where similar studies had been conducted (Table 5) in order to assess the intensity of deposition of PAHs with insoluble solids.

Comparable levels of total PAH concentrations (from 42 to 695.7 ng L^−1^) have been reported in Khabarovsk city [16]. The contamination in Barnaul (Southern Siberia) is slightly higher, with mean contents of PAHs varying from 179 to 4575 ng L^−1^. The contamination in Shelekhoy (Eastern Siberia) is much stronger, as the contents of PAHs there surpass the values for Tyumen by 1–2 orders of magnitude (up to 134,700 ng L^−1^), which can be explained by the influence of an aluminum plant [14]. Higher levels of contamination have also been observed in Swedish, Chinese, and Turkish cities. (see Table 5).

In some studies [12,76], the rates of PAH deposition with snow have been evaluated as per units of area. For comparative purposes, we converted our values of the total PAH deposition over the winter in Tyumen and obtained the rates of ∑14 PAH deposition from 1.0 to 65.5 μg m^−2^. In Moscow, the average rates of PM-phase PAH deposition are 45–57 μg m^−2^ in residential areas and 140–264 μg m^−2^ in transport zones [12]. Therefore, the rates of PM-phase PAH deposition in Tyumen are slightly lower than those in Moscow residential areas, but significantly lower than those in Moscow transport zones with intensive traffic. In Syktyvkar city, which is similar to Tyumen in terms of its geographic location and population numbers, the PAH deposition with snow has similar rates, i.e., 3.5–4.1 μg m^−2^ in the background area and 20.1–76.3 μg m^−2^ in the industrial zone [76].

The analytical results for benzo(a)pyrene (BaP), a ubiquitous carcinogenic pollutant, are of particular interest. The PM-phase BaP deposition in Tyumen had a rate of 2.7 ng m^−2^ per day (with a range from 0.12 to 7.3 ng m^−2^ per day). In Moscow, the PM-phase BaP deposition has daily rates from 2 to 28 ng m^−2^ in residential areas, 0.46 ng m^−2^ in the recreational zones, and 49.5 ng m^−2^ in the industrial zones [10]. Therefore, the benzo(a)pyrene deposition rate in Tyumen is close to that of the recreational zone of Moscow, but slightly lower than those of most residential areas in Moscow and significantly lower than those of the industrial zones of the Russian capital.

Therefore, the comparison demonstrates a low content of PAHs in the particulate matter of Tyumen snow. This is associated with a low dust load and the use of gas at local heat and power stations and for heating dwellings. Local meteorological conditions have consequences as well. In contrast to Eastern Siberia, where a winter anticyclone results in air blanketing, an anticyclone in Tyumen often gives way to air masses coming from the west and north, which results in scavenging of the atmosphere.

## 4. Conclusions

The mean total content of 14 PAHs in snowmelt water in the background area of Tyumen had a value of 6.2 ng L^−1^, which is lower than those of many unpolluted areas on Earth. This low content of PAHs was caused by the location’s long distance from pollution sources and an insignificant transfer of PM-phase PAHs from other regions. In the city of Tyumen, the mean content of particulate matter was five times higher (37.1 vs. 7.5 mg L^−1^) as compared to the background, and the mean total content of 14 PAHs was twenty times higher (122.8 vs. 6.2 ng L^−1^), with the contents of chrysene (8.8 vs. 0.11), benzo(k)fluoranthene (2.4 vs. 0.03) and benzo(a)pyrene (3.6 vs. 0.11 ng L^−1^) being increased by multiples of 78, 77, and 32, respectively. In contrast to the background areas, the share of HMW PAHs in the city grew due to the burning of motor fuels as their main energy sources. The highest content of PAHs was observed in the northern part of the city, near the roads that connect Tyumen with the northern areas of the region where oil and gas fields are situated. In the northern part of Tyumen, which is strongly impacted by motorized traffic, we identified an area dominated by PAHs of pyrogenic origin, i.e., resulting from liquid fuel combustion. The concentration of BaP in particulate matter of snow is by an order of magnitude higher than in soils, which makes snow the principal source of admission of this carcinogen into the soil during the thawing period. Therefore, the insoluble solids deposited with snow are one of the main sources of urban soil benzo(a)pyrene contamination. Therefore, we conclude on a practical basis that it is necessary to shovel snow and transport it to special storage areas.

Calculation of the diagnostic Flt/Pyr and LMW/HMW ratios produced the following results. Based on the LMW/HMW ratio, only 22% of samples, mainly taken in the transport zone, clearly originate from pyrogenic sources. The Flt/Pyr ratio also reveals a small share of samples (20%) with PAHs originating in the burning of oil products. A considerable number of samples were characterized by the value of Flt/Pyr ≈ 1, which is indicative of a combined effect of the burning of oil products and solid fuels (coal and firewood). Thus, analysis of spatial distribution, land-use effects, and diagnostic ratios showed that the PAHs in Tyumen were mainly from vehicle emissions and coal combustion.

Total contents of PM-phase PAHs decreased in the following order: transport zones > historical center > low-rise residential areas > industrial zones > high-rise residential areas > business and public facilities areas. Calculation of BaP toxic equivalent resulted in a somewhat different distribution of toxicity, decreasing in the following order: transport zones > low-rise residential areas > historical center > high-rise residential areas > business and public facilities areas > industrial zones. The greatest contributions to toxicity were made by BaP (53%), DahA (18%), and BbF (9.9%).

We made a comparison with other cities where similar studies have been conducted. This demonstrated that the total content of PAHs in Tyumen snow is relatively low and does not exceed the levels typical of Syktyvkar, Khabarovsk, and Moscow. Due to contamination from an operational aluminum plant, the content of PAHs in Shelekhov was higher by 1–2 orders of magnitude. The low content of PAHs in Tyumen is associated with a low dust load and the use of gas as the main fuel at local heat and power stations and for heating dwellings.

## Figures and Tables

**Table 1:** Physical characteristics of snow cover and dust deposition parameters in Tyumen.

Property	Tyumen (n = 46)	Background Area (n = 8)
Snow depth, cm	17–38 26	30–58 41
Snow density, g cm ^−3^	0.16–0.30 0.21	0.14–0.23 0.18
TDS, mg L^−1^	11.9–564.0 68.1	6.4–18.3 9.5
pH	5.9–8.1 6.3	4.2–5.5 4.7
Particulate matter content, mg L^−1^	9.4–121 37.1	4.1–10.9 7.5
Deposition flux values, mg m ^−2^ day ^−1^	5.4–94.3 20	2.4–8.3 5.5

n, number of sampling sites; numerator, min–max; denominator, arithmetic mean.

**Table 2:** Statistical summary of PM-phase PAH concentrations (ng L^−1^) in snow samples from Tyumen.

PAH Compounds	Background Area (n = 8)			Tyumen (n = 46)		
	Mean	Median	Min-Max	Mean	Median	Min-Max
Naphthalene (NaP)	1.36	0.80	nd–4.4	21.3	11.7	3.29–47.3
Fluorene (Flu)	0.18	0.18	nd–0.4	1.4	0.86	0.08–11.8
Phenanthrene (Phe)	2.19	2.20	1.55–3.25	26.4	14.0	4.69–198.5
Anthracene (Ant)	0.15	0.15	0.05–0.3	1.93	0.84	0.18–29.4
Fluoranthene (Flt)	0.90	1.00	nd–1.8	19.0	9.1	2.38–214.1
Pyrene (Pyr)	0.89	0.93	nd–2.4	16.4	7.8	0.92–211.3
Benzo(a)anthracene (BaA)	0.094	0.05	nd–0.3	2.2	1.6	nd–18.12
Chrysene (Chr)	0.11	0.00	nd–0.55	8.8	3.9	nd–201.6
Benzo(b)fluoranthene (BbF)	nd	0.00	nd	6.7	5.2	nd–62.57
Benzo(k)fluoranthene (BkF)	0.03	0.00	nd–0.1	2.4	1.6	nd–20.7
Benzo(a)pyrene (BaP)	0.11	0.08	nd–0.35	3.6	2.7	nd–22.6
Dibenzo(ah)anthracene (DahA)	0.13	0.00	nd–1.05	1.2	0.00	nd–15.3
Benzo(ghi)perylene (BghiP)	nd	0.00	nd	5.5	2.55	nd–46.7
Indeno[1,2,3-cd]pyrene (IcdP)	nd	0.00	nd	5.9	0.00	nd–41.8
∑14 PAHs	6.2	5.7	1.7–10.9	123	78.3	17.8–1019
∑LMW PAHs	4.78	4.5	1.7–8.15	70.1	36.0	13.3–575.2
∑HMW PAHs	1.38	1.2	nd–3.5	52.7	30.7	3.8–443.7

LMW: low-molecular-weight, HMW: high-molecular-weight, nd: not determined.

**Table 3:** Statistical indicators of PAH diagnostic values in different functional zones of Tyumen.

Land-Use Areas	Diagnostic Ratio	M	SD	Max
Historical center	LMW/HMW	1.9	1.5	4.0
	Flt/Pyr	1.3	0.3	1.7
Low-rise residential areas	LMW/HMW	1.3	0.4	1.7
	Flt/Pyr	1.4	0.3	1.7
High-rise residential areas	LMW/HMW	2.7	1.6	4.6
	Flt/Pyr	1.0	0.3	1.8
Business and public facilities areas	LMW/HMW	1.6	0.7	2.5
	Flt/Pyr	1.9	0.9	3.3
Industrial zone	LMW/HMW	1.9	0.8	2.7
	Flt/Pyr	1.2	0.4	1.8
Transport zone	LMW/HMW	1.0	0.3	1.6
	Flt/Pyr	1.2	0.3	1.9

M: mean, SD: standard deviation, Max: maximal value.

**Table 4:** Values of BaPeq in land-use areas of the Tyumen.

Land-Use Areas	BaPeq Values, Mean ± SD (Max)	Isomers Determining Toxicity ^×^
Historical center	6.0 ± 3.6 (11.1)	BaP_55_ DahA_22_ BbF_8_
Low-rise residential areas	6.2 ± 5.0 (14.5)	BaP_42_ DahA_37_ BbF_9_
High-rise residential areas	5.4 ± 7.7 (23.1)	DahA_41_ BaP_34_ IcdP_13_ BbF_5_
Business and public facilities areas	4.8 ± 3.7 (15.4)	BaP_65_ BbF_10_ IcdP_10_
Industrial areas	4.1 ± 3.2 (9.9)	BaP_55_ DahA_22_ BbF_8_
Transport areas	10.5 ± 7.2 (32.3)	BaP_55_ DahA_13_ BbF_11_ IcdP_9_
Tyumen in general	6.8 ± 5.5 (32.3)	BaP_53_ DahA_18_ BbF_10_ IcdP_9_

**^×^** subscript denotes the % contribution to total toxicity.

**Table 5:** A comparison of PAH content in snow from different locations.

City, Country	Concentration, ng L^−1^	Deposition, μg m^−2^	Source
Tyumen, Russia, ∑14 PAHs (particulate)	1.7–10.9 (background) 17.8–1018.9 (urban)	1.0–65.5	This study
Shelekhov, Russia, ∑13 PAHs (total)	28,600–134,700	-	[14]
Barnaul, Russia, ∑16 PAHs (total)	179–4575	-	[15]
Khabarovsk, Russia, ∑16 PAHs (total)	34.8–79.8 (background) 43–695.7 (urban)	-	[16]
Erzurum, Turkey, ∑18 PAHs (particulate)	23,820	-	[69]
Luleå and Umeå, Sweden, ∑16 PAHs (total)	2,720 (Luleå) 9,640 (Umeå)	-	[74]
Harbin, China, ∑16 PAHs (total)	10,700 (median value)	-	[75]
Changchun City, China, ∑16 PAHs (total)	26,600–36,900	-	[4]
Moscow, Russia, ∑16 PAHs (particulate)	-	45–57 (residential areas) 140–264 (traffic zone)	[12]
Syktyvkar, Russia, ∑13 PAHs (total)	-	3.5–4.1 background 20.1–76.3 industrial	[76]

Dash (-): no data.

## Data Availability

Not applicable.

## Supplementary Materials

The following supporting information can be downloaded at https://www.mdpi.com/article/10.3390/toxics10120743/s1, Table S1: Description of sampling sites.
