# Supplementary material for: Polycyclic Aromatic Hydrocarbons in the Snow Cover in the City of Tyumen (Western Siberia, Russia)
Source: Toxics. 2022 Nov 30;10(12):743. doi: 10.3390/toxics10120743 (PMC9785694; doi:10.3390/toxics10120743)
Supplement: Supplementary file 1 [file toxics-10-00743-s001.zip › toxics-2005622-supplementary.pdf]

Table S1. Description of sampling sites

Sample Name	Sample Number	Latitude	Longitude	Location	Collection Date
T1-2020	1	57,08900	65,62300	industrial zone	17.02.2020
T2-2020	2	57,09700	65,64200	high-rise residential area	17.02.2020
T3-2020	3	57,10100	65,60900	transport zone	17.02.2020
T4-2020	4	57,10900	65,61400	modern business zone	17.02.2020
T5-2020	5	57,10600	65,58800	high-rise residential area	17.02.2020
T6-2020	6	57,11400	65,57600	transport zone	17.02.2020
T7-2020	7	57,12861	65,57333	high-rise residential area	17.02.2020
T9-2020	8	57,14111	65,56139	modern business zone	17.02.2020
T10-2020	9	57,14500	65,58111	high-rise residential area	17.02.2020
T11-2020	10	57,15556	65,56028	transport zone	17.02.2020
T12-2020	11	57,146667	65,546389	low-rise residential area	17.02.2020
T13-2020	12	57,13000	65,53778	high-rise residential area	18.02.2020
T14-2020	13	57,12968	65,52968	low-rise residential area	18.02.2020
T16-2020	14	57,13472	65,50028	high-rise residential area	18.02.2020
T17-2020	15	57,13806	65,51417	modern business zone	18.02.2020
T18-2020	16	57,141111	65,5300	transport zone	18.02.2020
T19-2020	17	57,15306	65,61028	Industrial zone	18.02.2020
T20-2020	18	57,151944	65,653611	modern business zone	18.02.2020
T21-2020	19	57,11167	65,64611	industrial zone	18.02.2020
T22-2020	20	57,11667	65,62194	transport zone	18.02.2020
T23-2020	21	57,12139	65,59000	industrial zone	19.02.2020
T24-2020	22	57,133333	65,602778	industrial zone	19.02.2020
T26-2020	23	57,17750	65,60417	transport zone	19.02.2020
T27-2020	24	57,176500	65,651568	transport zone	18.02.2020
T28-2020	25	57,18694	65,62333	high-rise residential area	18.02.2020
T29-2020	26	57,19861	65,52806	transport zone	19.02.2020
T30-2020	27	65,548611	57,20083	historical center	19.02.2020
T31-2020	28	57,15028	65,53611	historical center	18.02.2020
T33-2020	29	57,15750	65,55139	historical center	19.02.2020
T34-2020	30	57,15694	65,52472	historical center	19.02.2020
T35-2020	31	57,15611	65,51306	historical center	19.02.2020
T36-2020	32	57,1475	65,524722	high-rise residential area	19.02.2020
T37-2020	33	57,18528	65,51444	low-rise residential area	19.02.2020
T38-2020	34	57,18750	65,46861	low-rise residential area	19.02.2020
T39-2020	35	57,16305	65,50138	industrial zone	19.02.2020
T40-2020	36	57,15889	65,48889	Industrial zone	19.02.2020
T41-2020	37	57,16611	65,46833	low-rise residential area	19.02.2020
T42-2020	38	57,15555	65,46638	low-rise residential area	19.02.2020
T43-2020	39	57,12056	65,54889	transport zones	19.02.2020
T44-2020	40	57,12111	65,50861	transport zones	19.02.2020
T45-2020	41	57,14528	65,45389	modern business zones	19.02.2020
T46-2020	42	57,14388	65,4850	transport zones	19.02.2020
T47-2020	43	57,14639	65,50722	modern business zones	19.02.2020
T48-2020	44	57,17333	65,53833	transport zones	19.02.2020
T49-2020	45	57,18361	65,55694	transport zones	19.02.2020
T50-2020	46	57,16861	65,57055	transport zones	19.02.2020
TB2-2020		56,837778	65,371667	Background area near Tyumen	20.02.2020
TB3-2020		56,867222	65,378611	Background area near Tyumen	20.02.2020
TB4-2020		57,081944	65,003889	Background area near Tyumen	20.02.2020
TB5-2020		57,055833	64,928056	Background area near Tyumen	20.02.2020
TB8-2020		57,30861	64,98472	Background area near Tyumen	20.02.2020
TB9-2020		57,315556	64,948333	Background area near Tyumen	20.02.2020
TB10-2020		57,35167	64,88722	Background area near Tyumen	20.02.2020
TB11-2020		57,22389	65,06278	Background area near Tyumen	20.02.2020
